# Twelve novel Italian cases of hypophosphatasia: challenging cases with an extremely variable phenotype

**DOI:** 10.3389/fendo.2026.1966666

**Published:** 2026-10-28

**Authors:** Giulia Casamassima, Tommaso Biagini, Flavia Pugliese, Anna Maria Grieco, Francesco Petrizzelli, Tommaso Mazza, Mauro Celli, Emanuela Scarano, Federica Tamburrino, Luciano Colangelo, Cristina Eller-Vainicher, Anda Mihaela Naciu, Gaia Tabacco, Giovanni Rossini, Francesca Pigliaru, Andrea Palermo, Silvia Morlino, Bartolomeo Augello, Antonio Petracca, Marco Castori, Alfredo Scillitani, Vito Guarnieri

**Affiliations:** 1 Inborn Errors of Morphogenesis Reseach Unit and Division of Medical Genetics, Fondazione Istituto di Ricerca a Carattere Scientifico (IRCCS)-Casa Sollievo della Sofferenza, San Giovanni Rotondo, Italy; 2 Computational Biology and Bioinformatics Unit, Fondazione Policlinico Universitario Agostino Gemelli Istituto di Ricerca a Carattere Scientifico (IRCCS), Rome, Italy; 3 Unit of Endocrinology, Fondazione Istituto di Ricerca a Carattere Scientifico (IRCCS) Casa Sollievo della Sofferenza, Foggia, Italy; 4 Rare Bone Diseases Center, AOU Policlinico Umberto I, Roma, Italy; 5 Pediatric Unit, Istituto di Ricerca a Carattere Scientifico (IRCCS) Azienda Ospedaliero-Universitaria di Bologna, Bologna, Italy; 6 Department of Medical and Cardiovascular Sciences, Sapienza University, Rome, Italy; 7 Unit of Endocrinology, Fondazione Istituto di Ricerca a Carattere Scientifico (IRCCS) Cà Granda Ospedale Maggiore Policlinico, Milan, Italy; 8 Unit of Endocrinology and Diabetes, Departmental Faculty of Medicine and Surgery, Campus Bio-Medico University of Rome, Rome, Italy; 9 Unit of Endocrinology, Azienda Ospedaliera-Universitaria of Cagliari, Cagliari, Italy

**Keywords:** ALPL, asymptomatic hypophosphatasia, enzymatic activity, functional assay, HPP, *in silico* prediction, TNSALP

## Abstract

**Context:**

Hypophosphatasia (HPP) is an inborn error of bone metabolism affecting the musculoskeletal and nervous systems. It is caused by monoallelic or biallelic pathogenic variants of the *ALPL* gene, which encodes the tissue non-specific alkaline phosphatase (TNSAP). Given its marked clinical variability ranging from prenatal presentations to symptoms onset in late adulthood, HPP often runs undetected with potential harms for affected individuals.

**Methods:**

Clinical, molecular, *in silico*, *in vitro*, and, when accessible, family studies were carried out on 12 HPP individuals with variable clinical presentations. Variants were classified according to the American College of Medical Genetics and Genomics recommendations.

**Results:**

Four cases were diagnosed as infantile, one as adult HPP. The other cases were completely asymptomatic (nr 4) or showed symptoms not strictly HPP-related (nr 3) but were all identified incidentally based on the biochemical low ALP value or severe bone fragility phenotype. Twelve “pathogenic o likely pathogenic variants” were identified including 9 missense (1 novel, 8 previously reported), 2 in frame indels and one multiexonic deletion. *In vitro* characterization of the seven point variants without previously described functional data confirmed a dramatic decreased enzymatic activity of some mutated enzymes.

**Conclusions:**

Improving the recognition of HPP is essential because its rarity and extreme variable presentation with many asymptomatic cases lead to misdiagnosis or delay. Increased clinical awareness and attention to persistently low ALP levels are crucial to achieve earlier and more accurate diagnosis.

## Introduction

Hypophosphatasia (HPP, OMIM #146300) is a hereditary bone metabolism disorder, affecting the musculoskeletal and nervous systems (1). It is caused by biallelic/monoallelic deleterious variants of the *ALPL* (NM_000478) gene, encoding the *tissue non-specific alkaline phosphatase* (*TNSALP)* (1). HPP is characterized by a wide clinical variability which is significantly but not uniquely associated with the levels of residual enzymatic activity (REA, 2). Accordingly, at least six different phenotypic classes are identified, depending on the age of onset and on the clinical severity and ranging from the lethal or benign perinatal, to the infantile (> 6 mths of age), to juvenile/childhood (6 mths to 18 ys) up to the mildest adult HPP (> 18 ys) or odontohypophosphalasia, the latter limited to dental manifestations (2). Literature also documents the existence of an increasing number of individuals presenting the biochemical profile of HPP (i.e, persistent low serum ALP levels, increased substrates, such as vitamin B6 and urinary phospho-ethanolamine) in absence of typical disease manifestations (e.g. fractures, musculoskeletal pain, premature loss of teeth) (3). While there is evidence that the most severe phenotypes are usually associated with biallelic inheritance, instances of dominant negative effects do not preclude early disease onset and aggressive disease course also in heterozygous individuals (4). However, diagnosis and prognosis of heterozygous individuals remains challenging given the possibility of an attenuated phenotype at presentation and an estimated prevalence of up to 1/6, 370 for dominant HPP in Europe (5).

To further complicate the diagnostic scenario, up to 80% of all *ALPL* variants identified to date are missense and often classified as of unknown clinical relevance (variants of uncertain significance; VUS) (6). The combination of (i) a presumably high rate of heterozygous individuals for *ALPL* variants in the general population, (ii) a preponderance of VUS among identified variants worldwide, and (iii) heterogeneity in inheritance pattern (autosomal recessive *vs* dominant) make the diagnostic confirmation of HPP a pitfall that often requires the support of multilayered investigations to reach a conclusive laboratory report. We report the clinical, genetic, *in vitro* and *in silico* studies of twelve novel patients with heterozygous/dominant HPP. Clinical presentations at referral were variable with infantile onset but diagnosis in adulthood, to adult onset, but mostly, biochemical diagnosis in asymptomatic individuals and subjects with atypical clinical presentation.

## Methods

### Patients’ enrollment

Patients were admitted at the outpatient clinics for “bone fragility and osteoporosis” of different tertiary care centres of Northern, Middle, Southern Italy and Sardinia. Eligibility criteria for requesting the genetic analysis of HPP was the blood ALP value (age and sex corrected) lower than the normal range measured at least twice, independently from the VitB6 (plasma pyridoxal 5′-phosphate, PLP value determined by HPLC) that was available in 10/12 subjects and higher (than the normal range) in 9. Exclusion criteria were the assumption of drugs affecting the bone turnover (bisphosphonates, denosumab), diseases interfering with the ALP activity/Vit B6 (hypomagnesaemia, hypozincemia, celiac disease, Wilson disease, hypothyroidism, hypoparathyroidism, multivitamin supplements). As reported in **Table 1**, some patients had bone pain, osteoporosis/low bone mass (defined as Tscore ≤ 2.5 for adults and Zscore ≤ -2.0 for children) and or multiple/single atraumatic fractures (defined as fractures derived from low energy trauma). At the admission, no other biochemical markers of bone turnover metabolism that could corroborate the final diagnosis (7) were available. Case (#8) has a normal ALP value and a border line VitB6 level: he was initially identified through differential diagnosis for severe bone fragility for a suspect of *Osteogenesis Imperfecta*. After the identification of the pathogenic ALP variant, he was included in the cohort.

**Table 1:** Clinical, biochemical and molecular data of the survey.

ID	Sex Age (ys)	AaD (ys) first symptoms	Delay of diagnosis (ys)	ALP (range, UI)	Vit B6 (7 – 18 μg/mL)	Symptomatic Asymptomatic Atypical	Clinical symptoms	HPP Class	Variant Nucleotide Protein	Novel (Y/N)	ACMG intepretations	WB (66/88 kDa)	REA (% WT)	Domain [ref (8)]	Missense3D Prediction/ Structural Damage	FoldX monomer dimer	Correlation		
																	Funct	In silico	Pheno
Patients carrying coding variants																			
#1	F 65	65	65	16 (35-104)	553	Asymptomatic	none	NAp	c.250G>A p.(Glu84Lys)	N	LP: PS1_Supp, PM2_Mod, PP2_Supp, PP3_Supp, PP4_Supp	DE	0	homodimer interface^a^	Neutral NA	2.97 5.25	Yes		No
#2	F 59	59	59	36 (35-104)	74	Asymptomatic	none	NAp	*c.407G>T* *p.(Arg136Leu)*	*N*	*LP: PS4_Str, PM2_Mod, PP2_Supp, PP3_Supp, PP4_Supp*	*DE*	*50*	*active site valley*	*Neutral* *NA*	*1.93* *3.44*	*Yes*		No
#3	F 57	57	57	25 (45-117)	NA	Asymptomatic	LBM*	NAp	c.920C>T p.(Pro307Leu)	N	P: PS1_Str, PS3_Supp, PM2_Mod, PP2_Supp, PP3_Supp, PP4_Supp	LoE	0	Ca^2+^?	Neutral NA	4.63 8.72	Yes		No
#6	M 9	3 growth delay	6	83 (86-315)	264.7	Atypical	growth delay	Inf	c.500C>T p.(Thr167Met)	N	LP: PS1_Str, PM2_Mod, PP2_Supp, PP3_Supp, PP4_Supp	NE	10	active site vicinity?	Neutral NA	0.1 1.13	No		
#7	M 7	4 NDD	3	89 (90-273)	244.5	Atypical	neural, motor, demabulation and growth delay, hyperlaxity, muscular hypotonia	Inf	c.1471G>A p.(Gly491Arg)	N	LP: PS1_Str, PM2_Mod, PP2_Supp, PP3; Supp, PP4_Supp	LoE	50	homodimer interface	Damaging Gly in a bend	-1.07 -1.54	No		
#8	M 16	7 fractures	9	115 (51-171)	21	Atypical	MF (radius, ulna, skull), OstP, edentulia, LBM	Inf?	c.815G>A p.(Arg272His)	N	LP: PS1_Str, PM2_Mod, PP2_Supp, PP3_Supp, PP4_Supp	DE	20	NA	Neutral NA	4.11 10.32	Yes		
#9	M 42	6 fractures	36	33 (40-129)	13.5	Symptomatic	MF (tibia, wrist, phalanxes), OstP, paresthesia	Inf	**c.521C>T** **p.(Pro174Leu)**	**Y**	**LP: PM2_Mod, PM5_Supp, PP2_Supp, PP3_Supp, PP4_Supp**	**DE**	**10**	**active site vicinity?**	**Neutral** **NA**	**1.55** **2.53**	**Yes**		
#11	F 57	10 fractures	42	25 (35-104)	25	Symptomatic	MF (pediatric age), OstP, edentulia, kyphoscoliosis, OP	Inf	*c.1144G>A* *p.(Val382Leu)*	*N*	*LP: PS1_Str, PM2_Mod, PP2_Supp, PP3_Supp, PP4_Supp*	*NP*	*2.7*	*active site vicinity*	*Neutral* *NA*	*0.65* *1.38*	*No*		
#12	F 68	68 fractures	18	21 (40 - 150)	24.5	Symptomatic	MF (humerus and ankle)	Ad	*c.1277G>A* *p.(Gly426Asp)*	*N*	*LP: PS1_Str, PM2_Mod, PP2_Supp, PP3_Supp, PP4_Supp*	*DE*	*10*	*crown*	*Damaging* *Disallowed phi/psi*	*3.3* *5.83*	*Yes*		
Patients carrying in frame deletions or the large genomic deletion																ΔΔG	Correlation		
																	Funct	In silico	Pheno
#4	F 39	39	39	17 (46-116)	104	Asymptomatic	LBM*	NAp	c.1318_1320delAAC p.(Asn440del)	N	LP: PS1_Supp, PM2_Mod, PM4_Mod, PP4_Supp	DE	10	collagen binding	NA NA		No		
#10	F	10 fractures	43	40 (45-117)	76.6	Symptomatic	OstP, hypermobility, fractures (wrist, phalanx)	Inf	c.984_986del p.(Phe328del)	N	LP: PS3_Supp, PM2_Mod, PM4_Mod, PP4_Supp	DE	10	others	NA NA	1.85	Yes		No
#5	F 64	54 spondilo-osteoathritis	64	17 (46-116)	NA	Atypical	spondilo-osteoathritis	NAp	chr1:g.(21836992_21880592)_(21887592_21889695)del (hg19)	NA	P: PVS1_Str, PM2_Mod, PP4_Supp	NAp	NAp	NAp	Nap Nap	-4.2	No		

*identified after the biochemical and genetic diagnosis; AaD, Age at Diagnosis; AaO, Age at Onset; NDD, neuro developmental disorder; MF, multiple fractures; OP, osteoporosis; LBM, low bone mass; OstP, osteoarticular pain; Nap, not applicable; NA, not available. Functional and modeling data of the TNSALP coding sequence variants: in bold the novel variant: https://alplmutationdatabase.jku.at/, last release 2026, August 12ve; in italic the variants for whose functional data were derived from literature; WB, Western Blot; NE, Normal Expression; LoE, Loss of Expression; DE, Down Expression; REA, Residual Enzymatic Activity; please note that for “inframe-deletions” the prediction tools were not applied (see the Methods); NAp, not applicable; NA, not available; NP, not performed.

### NGS and functional assays

DNA was extracted from blood withdraw and subjected to a massive parallel sequencing. NGS protocol, variants filtering, prioritization and interpretation as well Western Blot, Colorimetric Assay and 3D modelling were described in the **Supplementary Materials**.

### TNSALP variants tested

Functional assays were done on the novel variant [p.(Pro174Leu)] and on those already reported but not functionally tested: p.(Thr167Met), p.(Arg272His), p.(Pro307Leu), p.(Phe328del), p.(Asn440del), p.(Gly491Arg) (6).

## Results

### Patients

Four probands (#9 to #12), were identified in adulthood (42, 53, 57, 68 years) but they yet reported first symptoms at 6, 10, 10 and 68 years, respectively. The clinical picture combined with persistently low ALP level, led *ab initio* in 3 (#9 to #11) to the diagnosis of childhood HPP while pt #12 was diagnosed as adult HPP, further confirmed by the genetic analysis.

Four adults (#1 to #4, 39–65 ys) underwent a more thorough clinical examination because of repeated low serum ALP levels determined during routine screening, in apparent absence of other overt clinical signs. After the biochemical finding, osteoporosis/low bone mass was discovered in #3 (L1-L4-Tsc = -1, 6; Zsc = -0, 1; FN-Tsc = -2, 5; Zsc = -1, 3; FT-Tsc = -2, 5; Zsc = -1, 6) and #4 (L1-L4-Tsc = -1, 2; Zsc = -0, 3; FN-Tsc = -2, 7; Zsc = -1, 5; FT-Tsc = -2, 5; Zsc = -1, 8), while the others (#1 and #2) remain with no other symptom. Molecular testing was requested and the diagnosis was made after the genetic confirm and segregation analysis was enlarged to available relatives, confirming the inheritance of the disease (**Figure 1**).

**Figure 1:**
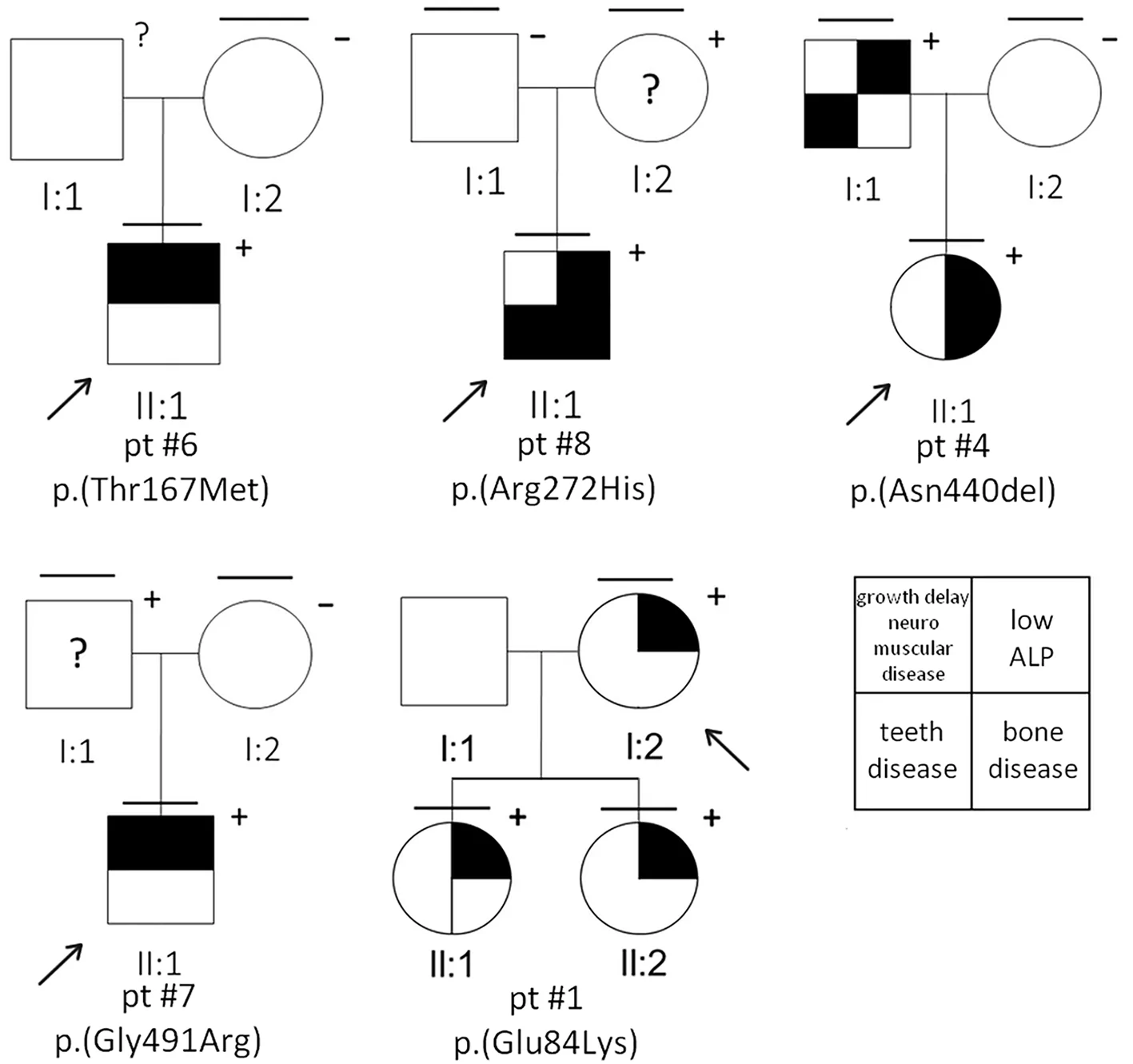
Pedigree of some families for whose segregation analysis was performed. “+”, carrier; “-”, not carrier; solid line, tested for segregation analysis; “?”, not tested for segregation analysis.

Three subjects (#5 to #7) presented a clinical picture unrelated to HPP. Indeed, patients #6 and #7 were affected at an early age (3 and 4 ys) by growth (#6) or neuromotor/developmental delay, short stature, generalized hyperlaxity (#7). Patient #5 was affected in the previous 10 ys only by spondilo-osteoarthirtis (that is not among the common HPP clinical signs) (8, 9); after the finding of persistent low ALP value, in all, molecular analysis was requested and they were diagnosed as infantile or adult HPP, after the identification of the deleterious ALPL variant.

Finally, patient #8 was likewise atypical in his clinical presentation since, although he had a normal ALP and slightly high Vit B6 level, he manifested the more classic HPP signs (multiple fractures, edentulia, osteopenia and low bone mass) so that a suspect of osteogenesis imperfecta raised. However, molecular testing for a specific panel of bone fragility related genes resulted in a heterozygous “likely pathogenetic” ALP variant, while screening on OI related genes resulted negative for both single nucleotide variants and copy number variations (CNV).

### NGS and MLPA

Nine missense variants [*p.(Glu84Lys), p.(Arg136Leu), p.(Thr167Met), p.(Pro174Leu), p.(Arg272His), p.(Pro307Leu), p.(Val382Ile), p.(Gly426Asp), p.(Gly491Arg)], 2 indel [p.(Phe328del), p.(Asn440del)*] and 1 intragenic *copy number variation* (CNV) [*chr1:g.(21836992_21880592)_(21887592_21889695)del (hg19)*] (**Figure 2**) were identified all in heterozygosity in the twelve patients. The *p.(Pro174Leu)* and the deletion of exons 2-3–4 are novel, while other missense and indel variants were reported (6).

**Figure 2:**
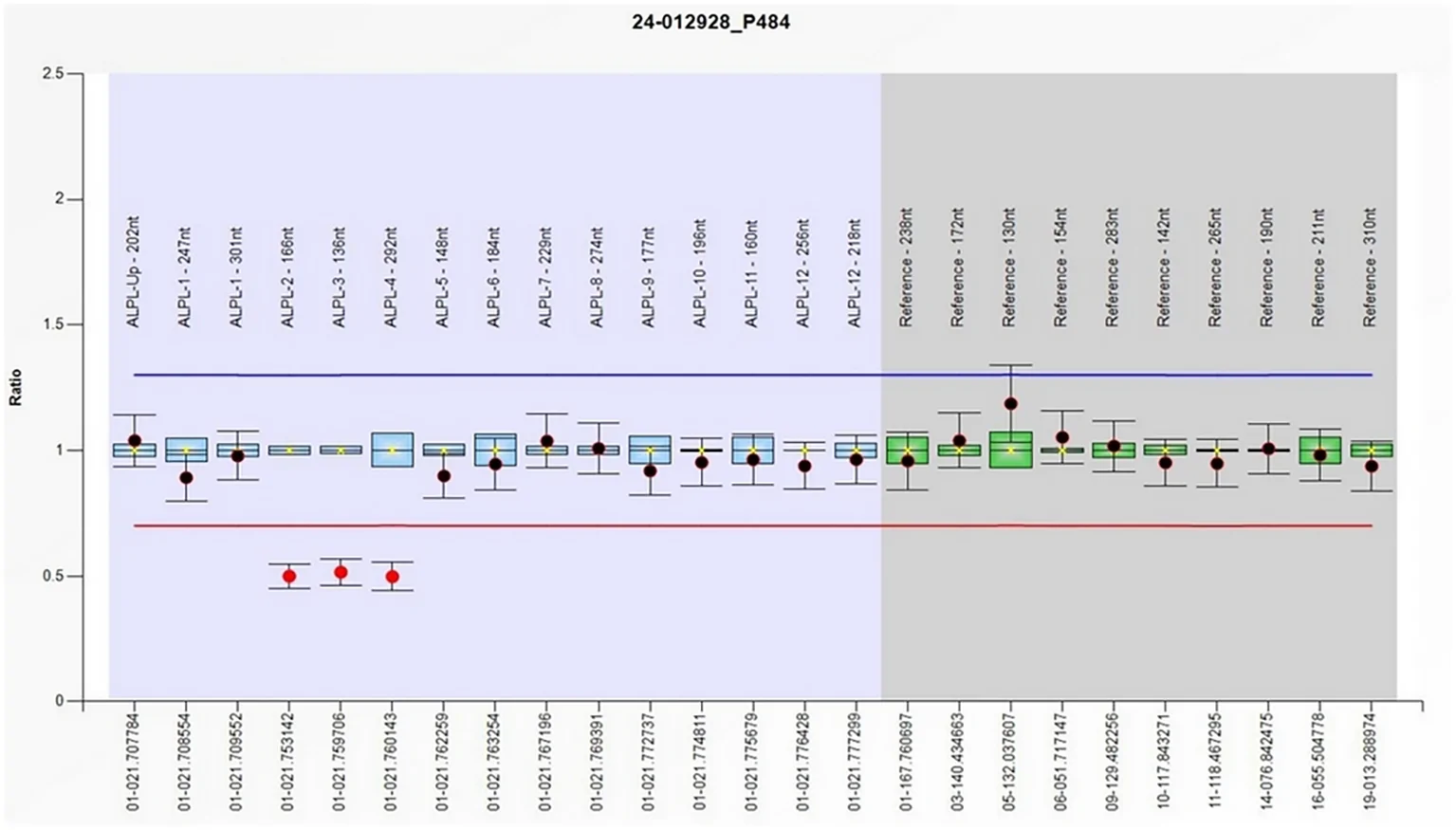
Boxplot chart showing the deletion of exon 2–4 in patient #5.

Following the criteria released by the ACMG (10), all the variants were classified as “Likely Pathogenic” except for the *p.(Gly491Arg)* and the multiexonic deletion that were classified as “Pathogenic” (**Table 1**). Segregation analysis was done on available familial members. Interestingly, for two asymptomatic cases, lack of clinical symptoms was confirmed in both the daughters of case #1, while for the second (#4), the father was found as affected by previous dental abscesses and then he received the diagnosis after the daughter, at 74 ys (data not shown).

### Expression of the differential bands

Into the cell, TNSALP is present in three forms: a cytoplasmic of 58 kDa, an ER-located glycosylated of 66 kDa and a Golgi-located mannosidated of 88 kDa (11). In **Figure 3a**, the 66 and 88 kDa forms of the WT/Mut proteins were shown. While the *p.(Pro174Leu)* mutant was apparently normally expressed, all the mutants showed a strong decreased expression of both the bands up no more than the 50% compared to WT with 4 mutants barely expressed [*p.(Gly491Arg), p.(Asn440del), p.(Arg272His) and p.(Thr167Met)*] (**Figure 3b**).

**Figure 3:**
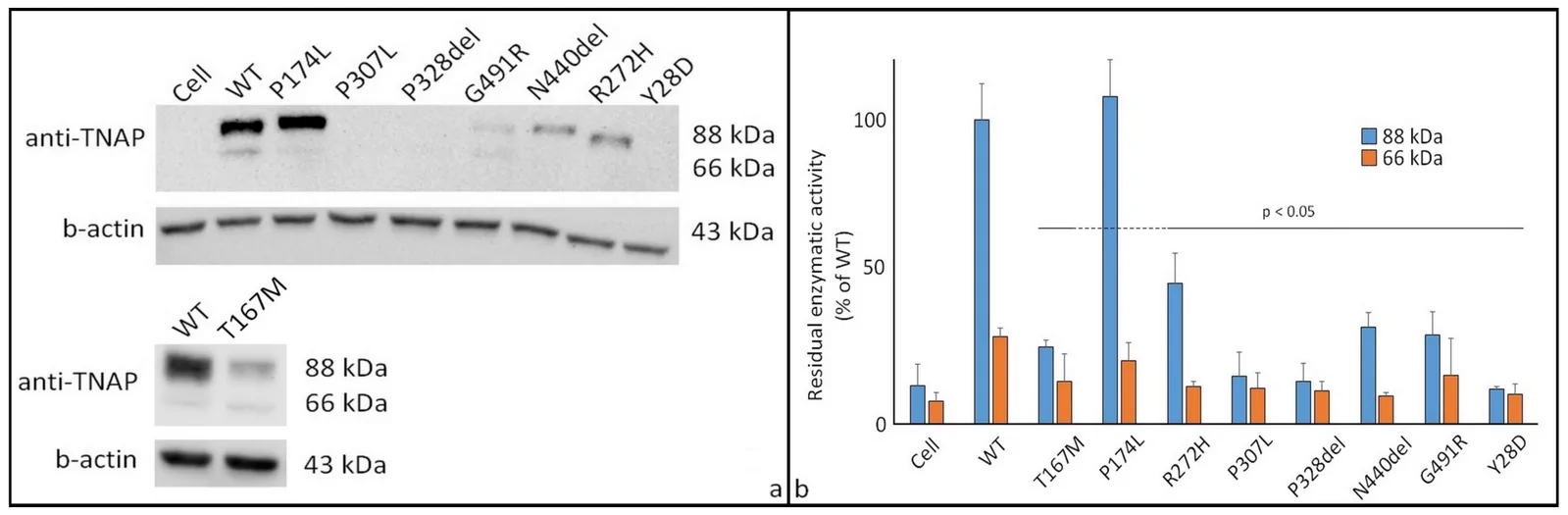
**(a)** western blot showing the expression of 66 and 88 kDa bands and **(b)** quantification as relative expression (% of WT) plotted in the histograms.

### Enzymatic activity

The colorimetric assay confirmed a clear decreased enzymatic activity of mutants ranging from the ~30% for the *p.(Arg272His)* to the ~50% for the *p.(Gly491Arg)*, while the remaining variants were almost totally inactive, showing a REA comparable with not transfected cells and with the internal negative control [*p.(Tyr28Asp)*] (**Figure 4**).

**Figure 4:**
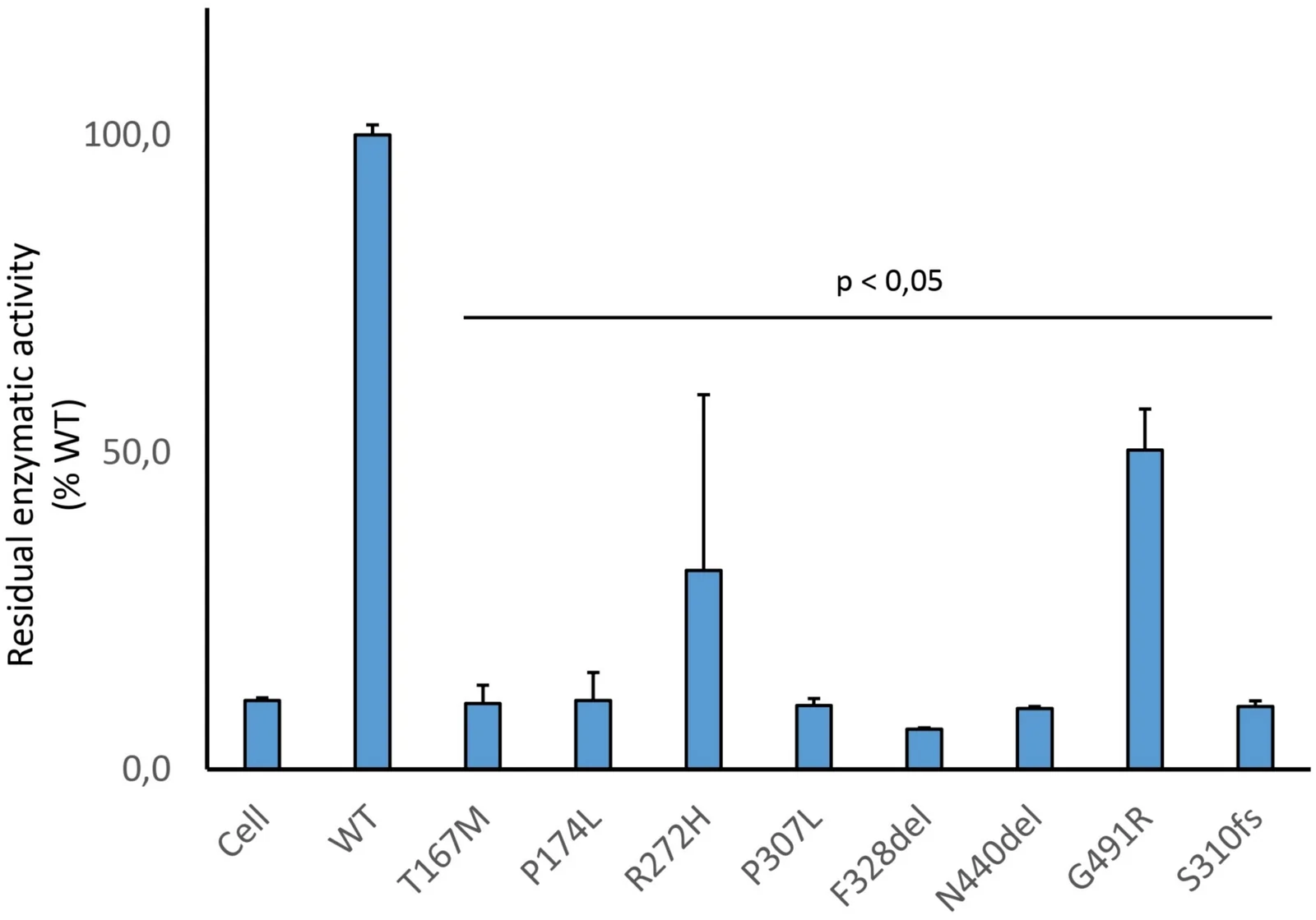
Residual Enzymatic Activities (REA) are plotted as relative expression (% of WT) for each mutant.

### 3D prediction

The structural impact of the 5 missense variants was assessed *in silico* through two approaches: Missense3D, which identifies alterations in structural features introduced by the amino acid substitution and FoldX, which estimates the variation in protein thermodynamic stability (ΔΔG, expressed in kcal/mol; values > 0.61 are indicative of a significant destabilizing effect).

Missense3D classified *p.(Gly491Arg)* as “damaging”, due to the substitution of a glycine located in a structural bend, whereas the remaining variants [*p.(Thr167Met), p.(Pro174Leu), p.(Arg272His), p.(Pro307Leu)*] were predicted to be neutral with respect to local structural alterations. Conversely, FoldX analysis revealed a different picture in terms of stability: a marked destabilizing effect was observed for *p.(Pro307Leu)* (ΔΔG = +4.63), *p.(Arg272His)* (ΔΔG = +4.11), *p.(Pro174Leu)* (ΔΔG = +1.55), whereas *p.(Thr167Met)* showed a negligible impact on stability (ΔΔG = +0.10) and *p.(Gly491Arg)* a slight stabilizing effect (ΔΔG = −1.07).

The two approaches proved to be complementary: Missense3D highlighted a localized structural impairment restricted to *p.(Gly491Arg)*, while FoldX identified a significant energetic destabilization for *p.(Pro174Leu), p.(Arg272His), p.(Pro307Leu)*, consistent with the marked reduction in residual enzymatic activity and expression levels observed *in vitro*. These methods were not applied to the in-frame deletions; however, the region ecompassing each deletion is shown in **Figure 5**.

**Figure 5:**
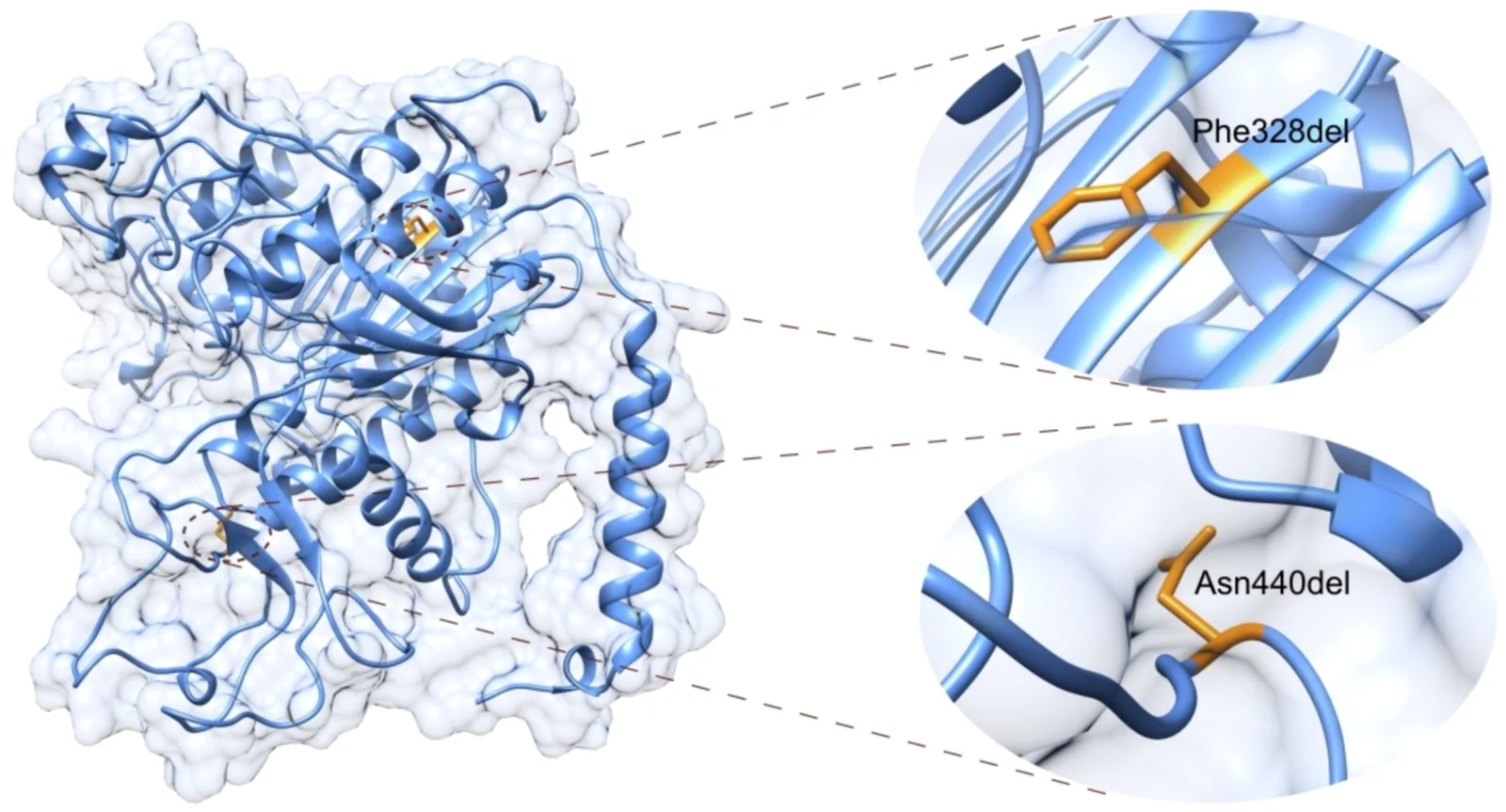
3D structure of *ALPL* monomer obtained through molecular modeling. The locations of Phe328del and Asp440del mutations are mapped on the wild-type structure and highlighted in orange; details are shown in the circular insets.

## Discussion

Diagnosing hypophosphatasia in clinically normal or minimally symptomatic individuals is challenging due to the absence of overt skeletal or dental manifestations. These patients may present only with persistently low serum ALP levels, which are often incidental findings and easily disregarded. Without clear clinical signs, HPP may remain unrecognized leading to a severe delay in patient’s management and, sometimes, to dangerous treatments. Moreover, in absence of specific longitudinal studies on such patients, it is not known whether, in the future, they will develop or not a mild (full) spectrum of the disease, although a recent work demonstrated as in frank HPP subjects, clinical features might accumulate over time, suggesting a gradual impairment of the clinical conditions (12).

Here we present 12 novel HPP cases, all caused by a heterozygous deleterious genetic variant, 11 of the coding sequence and 1 large genomic deletion of the *ALPL* gene. In four patients (#9 to #12), the diagnosis of HPP was made on the base of biochemical and clinical evaluation consisting of persistent low ALP value with multiple atraumatic fractures, musculoskeletal pain, edentulia and arthralgia; in these patients, the molecular finding followed and confirmed the clinical suspicious. In contrast, the remaining cases were almost completely asymptomatic (#1 to #4) or complained of nonspecific HPP-related symptoms (#5 with spondyloarthritis, #6 with growth retardation, #7 with neuromotor/developmental delay, short stature, and generalized hyperlaxity) or manifested all the typical signs of HPP (#8, multiple fractures, edentulia, osteopenia/low bone mass) but with an unexpected normal ALP value. In all these atypical patients, identified incidentally, during routine biochemical screening or because of a severe bone fragility phenotype (#8), genetic screening and molecular confirm were necessary to reach the final diagnosis.

Interestingly, the delay in disease recognition was not limited to atypical cases, where it would be expected (range 3–65 ys). Indeed, even for patients who manifested a suggestive clinical spectrum, the definitive molecular diagnosis was achieved only after a delay ranging from 9 up to about 40 years from disease onset (**Table 1**). This highlights the need for a deeper understanding of the pathophysiology and clinical presentation of the disease, in order to identify as early as possible subjects who should be referred for specific therapy and follow-up.

From a molecular perspective, we asked whether the new variant, as well as other known variants not previously studied, were indeed functionally impaired, given the atypical or asymptomatic state of some patients.

### E84K-P307L-T167M-N440del

Mutants showed consistent results being all down expressed at WB (DE/LoF = ~0-25%) (13), with a REA comparable with the signal noise (~0-10%) (13). However, the main point to take into account is the absence of clinical symptoms although a REA almost negligible for patients #1, #3 and #4.

### R272H-P328del

Also for these mutants, a perfect correlation between the WB and REA results were provided. Instead the WB show that the R272H was down expressed and partially enzymatic inactive (DE = ~40%, REA = ~30%) while for the P328del, no bands were visible at the WB with a consistently loss of enzymatic activity (REA = ~0-10%).

### R136L-G426D-V382L

Data were derived from literature with low discrepancy between the results (DE = ~50% at the WB, REA = 0-30%, for the V382L only the REA was available = ~0-10%, database).

### P174L

At the Western Blot, this mutant seemed not to be impaired in the expression (NE = 100%), but it was likewise enzymatically not functional (REA = ~0-10%). This inconsistency might be considered apparent: instead, the P174 is located close to the active site and it is likely that the amino acid change interferes with catalytic activity rather than affecting protein stability. Should further studies would confirm this hypothesis the functional results would be in line with the phenotypic presentation (**Table 1**).

### G491R

Functional results are not completely in line, since the mutant is down expressed at the WB (DE = 0-25%), while it showed a not negligible REA (=~50%).

Given the lack of overt symptomatology of asymptomatic patients (nr = 4) or the atypical presentation in others (nr = 4), a comparison of *in vitro* and in silico results with clinical presentation was attempted only for the remaining 4 symptomatic patients with a possible trend only for case #12 (DE = ~50%, REA = ~10%, FoldX monomer = 3.3, dimer = 5.83). For other variants, although they were confirmed as almost totally inactive by functional and ‘in silico’ tests, it is not known why they apparently did not cause a corresponding clinical picture. This could be due to other physiological compensating factors that may have attenuated the overall phenotype presentation (3), or, conversely, the lack of likely genetic alterations of genomic regions usually not screened in the routine diagnostic such as large intronic sequences, 5’ or 3’ untranslated regions or to rare epigenetic mechanisms (3) that could contribute to the compromised clinical status.

An additional possible explanation can be found into the data already available in the official *ALPL* database (6). Indeed, three variants *[(p.(Arg136Leu), p.(Pro307Leu) and p.(Asn440del)*] were identified in compound heterozygosity in HPP cases variably classified (adult, childhood and odonto-HPP), while only the *p.(Pro307Leu)* was also reported in heterozygosity in an AD adult form (6). This may suggest that: 1) in heterozygosity, these aminoacidic changes would not be able to impact from a clinical point of view but rather only the biochemical phenotype and 2) they do not possess any dominant negative effect. It is worthy to note that 3 out of 4 available relatives, carriers of these corresponding variants, were confirmed to be totally asymptomatic. Anyway, further studies on larger cohorts are needed to support this hypothesis.

Case #8 deserves a separate comment, being atypical in his presentation yet one opposite to that observed in the other patients. He was suspected to have an osteogenesis imperfecta-like form due to the severe clinical phenotype (multiple fractures, edentulia, osteopenia/low bone mass), while maintaining a persistently normal ALP value and NGS-based differential diagnosis identified a well-established pathogenic *ALPL* variant. The patient inherited the variant from the mother who was asymptomatic (**Figure 1**), but a variable penetrance is not uncommon in HPP families (1) and literature data reported the aminoacidic change other 3 times, in two homozygote/compound heterozygote subjects and once as single variant in an AD case, for whom biochemical ALP data were unavailable (6). This additional atypical phenotypic presentation is not infrequent, at least in our experience. Instead, we previously reported 2 patients with a clinical HPP phenotype, a known heterozygous pathogenic *ALPL* variant, but normal ALP levels (14). The reasons for this discordance remain unclear, as does whether a true hypophosphatasemia may develop later. Certainly, these cases further complicate the clinical scenario associated to the disease.

With regard possible treatment approaches, enzyme replacement therapy (ERT) with recombinant enzyme (18) could be used for symptomatic patients (#9 - #12) who have frank biochemical as skeletal and systemic symptoms. The same approach could be used for patient #8, who is symptomatic but carrier of a pathogenetic variation of the ALPL gene, even if the ALP activity appears to be within the normal range, however, under a rigorous clinical and biochemical follow-up to assess the therapeutic benefit.

Apart these “edge cases”, a persistent low ALP value would remain the main indicator to ask for genetic analysis. However, the genetic counseling might represent a challenge for subjects with biochemical HPP (14–17) owing to the aforementioned characteristics of multiple inheritance modes (AD, AR and DNE), extreme phenotypic variability and widespread prevalence of (hypomorphic)? *ALPL* alleles in the general population (up to 1/6330 in Europe) (5). Certainly, the genetic counseling as the segregation analysis should always be offered the identified “carriers” confirmed as having hypophosphatasemia and managed as “patients with subclinical HPP” (19), avoiding antiresorptive treatments (20) as precautionary measure, while acknowledging the possibility that they might also never develop a clinical counterpart of the disease.

We acknowledge that our study has certain limitations: in addition to the small sample size, for most patients, biochemical data (vitamin B6, Z-scores, T-scores) were unavailable. Furthermore, we cannot rule out potential selection or referral bias of the Italian tertiary care centers involved, nor differences in clinical assessment approaches. Finally, longitudinal follow-up of asymptomatic patients is required to complete their clinical description and to verify whether they will actually develop a clinical overt HPP spectrum. However, others reported similar results (3) and our findings are pretty in line. A recent retrospective analysis identified 43 genetically confirmed HPP cases, either AD or AR, showing only a biochemical phenotype (low ALP activity and high Vitamin B6) (3). Among these, now we can include the *p.(Glu84Lys)* variant previously characterized by our group (12), and here reported in a novel family, which still cause only a reduction in ALP activity, without clinical symptoms (**Table 1**), either in the relatives.

## Conclusions

Our work enlarges the number of subjects with asymptomatic HPP (3), a condition that seems more common than expected. This emphasizes the need for a multidisciplinary approach and a deeper understanding of HPP-related pathology for a quicker identification. We also expand the knowledge of the ALPL variant’s impact on protein structure and enzymatic function; although further molecular studies are needed to fully understand the lack of any clinical effect despite a clear functional impairment of the mutant. Undoubtedly, the increasingly widespread use of WES in routine diagnostic practice will soon increase the number of identified individuals carrying an *ALPL* variant. The availability of biochemical and/or clinical data on such subjects would greatly enhance our understanding of the disease’s pathophysiology and on likely progression from a just biochemical to a full clinical form.

## Data Availability

All relevant data is contained within the article: The original contributions presented in the study are included in the article/**Supplementary Material**, further inquiries can be directed to the corresponding author.

## References

[1] Villa-Suárez JM García-Fontana C Andújar-Vera F González-Salvatierra S de Haro-Muñoz T Contreras-Bolívar V . Hypophosphatasia: a unique disorder of bone mineralization. Int J Mol Sci. (2021) 22:4303. doi: 10.3390/ijms22094303 33919113 PMC8122659

[2] Mornet E . Hypophosphatasia. Orphanet J Rare Dis. (2007) 2:40. doi: 10.1186/1750-1172-2-40 17916236 PMC2164941

[3] Montero-Lopez R Farman MR Högler F Rehder C Malli T Webersinke G . Biochemical phenotype of hypophosphatasia in asymptomatic individuals carrying *ALPL* variants. J Bone Miner Res. (2026) 41:259–69. doi: 10.1093/jbmr/zjaf124 41042986 PMC13017406

[4] Fauvert D Brun-Heath I Lia-Baldini AS Bellazi L Taillandier A Serre JL . Mild forms of hypophosphatasia mostly result from dominant negative effect of severe alleles or from compound heterozygosity for severe and moderate alleles. BMC Med Genet. (2009) 10:51. doi: 10.1186/1471-2350-10-51 19500388 PMC2702372

[5] Mornet E Yvard A Taillandier A Fauvert D Simon-Bouy B . A molecular-based estimation of the prevalence of hypophosphatasia in the European population. Ann Hum Genet. (2011) 75:439–45. doi: 10.1111/j.1469-1809.2011.00661.x 21488855

[6] ALPL mutation database. Available online at: https://ALPLmutationdatabase.jku.at/ (Accessed August 12, 2026).

[7] Bertoldo F Tripepi G Zaninotto M Plebani M Scillitani A Varenna M . Possible role of bone turnover markers in the diagnosis of adult hypophosphatasia. J Bone Miner Res. (2025) 40:79–86. doi: 10.1093/jbmr/zjae177 39498489 PMC11983269

[8] Reis FS Lazaretti-Castro M . Hypophosphatasia: from birth to adulthood. Arch Endocrinol Metab. (2023) 67:e000626. doi: 10.20945/2359-3997000000626 37249457 PMC10665056

[9] Khan AA Brandi ML Rush ET Ali DS Al-Alwani H Almonaei K . Hypophosphatasia diagnosis: current state of the art and proposed diagnostic criteria for children and adults. Osteoporos Int. (2024) 35:431–8. doi: 10.1007/s00198-023-06844-1 37982857 PMC10866785

[10] Richards S Aziz N Bale S Bick D Das S Gastier-Foster J . Standards and guidelines for the interpretation of sequence variants: a joint consensus recommendation of the American College of Medical Genetics and Genomics and the Association for Molecular Pathology. Genet Med. (2015) 17:405–24. doi: 10.1038/gim.2015.30 25741868 PMC4544753

[11] Shibata H Fukushi M Igarashi A Misumi Y Ikehara Y Ohashi Y . Defective intracellular transport of tissue-nonspecific alkaline phosphatase with an Ala162→Thr mutation associated with lethal hypophosphatasia. J Biochem. (1998) 123:968–77. doi: 10.1093/oxfordjournals.jbchem.a022032 9562633

[12] Szabo SM Tomazos IC Petryk A Powell LC Donato BMK Zarate YA . Frequency and age at occurrence of clinical manifestations of disease in patients with hypophosphatasia: a systematic literature review. Orphanet J Rare Dis. (2019) 14:85. doi: 10.1186/s13023-019-1062-0 31023354 PMC6485115

[13] Casamassima G Grieco AM Biagini T Buono G Cinque L Pugliese F . Functional and in silico characterization of *ALPL* gene variants reveals genotype–phenotype correlations in Italian hypophosphatasia patients. Cells. (2025) 14:1768. doi: 10.3390/cells14221768 41294821 PMC12651241

[14] Cinque L Pugliese F Salcuni AS Trombetta D Battista C Biagini T . Clinical and molecular description of the first Italian cohort of 33 subjects with hypophosphatasia. Front Endocrinol (Lausanne). (2023) 14:1205977. doi: 10.3389/fendo.2023.1205977 PMC10433156 37600704

[15] Kumar S Duncan EL Hayes L Berman Y Clifton-Bligh RJ De Sousa SMC . Heritable metabolic bone disorders: a guide to current genetic testing and clinical management for adult endocrinologists. Pathology. (2026) 58:189–98. doi: 10.1016/j.pathol.2025.12.001 41558912

[16] Sperelakis-Beedham B Taillandier A Domingues C Guberto M Colin E Porquet-Bordes V . Utility of genetic testing for prenatal presentations of hypophosphatasia. Mol Genet Metab. (2021) 132:198–203. doi: 10.1016/j.ymgme.2021.01.009 33549410

[17] Huggins E Ong R Rockman-Greenberg C Flueckinger LB Dahir KM Kishnani PS . Multigenerational case examples of hypophosphatasia: challenges in genetic counseling and disease management. Mol Genet Metab Rep. (2020) 25:100661. doi: 10.1016/j.ymgmr.2020.100661 33101980 PMC7578550

[18] Bowden SA Foster BL . Alkaline phosphatase replacement therapy for hypophosphatasia in development and practice. Adv Exp Med Biol. (2019) 1148:279–322. doi: 10.1007/978-981-13-7709-9_13 31482504

[19] Kishnani PS Seefried L Ozono K Martos-Moreno GA Rockman-Greenberg C Fowler D . The Global Hypophosphatasia Registry: lessons learned from a decade of real-world data. Orphanet J Rare Dis. (2025) 20:626. doi: 10.1186/s13023-025-04129-w 41286962 PMC12751868

[20] Rassie K Dray M Michigami T Cundy T . Bisphosphonate use and fractures in adults with hypophosphatasia. JBMR Plus. (2019) 3:e10223. doi: 10.1002/jbm4.10223 31687651 PMC6820463

