# Supplementary material for: Twelve novel Italian cases of hypophosphatasia: challenging cases with an extremely variable phenotype
Source: Front Endocrinol (Lausanne). 2026 Oct 28;17:1966666. doi: 10.3389/fendo.2026.1966666 (PMC13751422; doi:10.3389/fendo.2026.1966666)
Supplement: Supplementary file 1 [file DataSheet1.pdf]

Supplementary Materials

NGS. DNA was extracted from blood and sequenced using an NGS-targeted panel (Sure Select gene panel; Agilent Technologies, Santa Clara, Ca, USA) designed for genes known to be associated with syndromic and non-syndromic diseases associated to bone fragility and density alterations: *ADAMTS20, AGBL2, ALPL, ANO5, ASCC1, B3GALT6, B4GALT7, BMP1, CA, CCDC134, CHST3, CLCN7, COL1A1, COL1A2, CREB3L1, CRTAP, CSF1R, CTSK, FAM111A, FAM46A, FGF23, FKBP10, FKBP14, GALNT3, GORAB, GPR68, HSPG2, IFITM5, KDELR2, KL, LEPRE1, LIFR, LRP5, LRRK1, MBTPS2, MESD, MITF, OSTM1, P4HB, PFN1, PLEKHM1, PLOD1, PLOD2, PLOD3, PLS3, PPIB, RELA, RUNX2, SAMD9, SEC16B, SEC24D, SERPINF1, SERPINH1 SGMS2, SLC29A3, SLC34A1, SLC9A3R1, SNX10, SP7, SPARC, SQSTM1, TCIRG1, TMEM38B, TNFRSF11A, TNFSF11, TRAF6, ,UNC45A, USB1, VAV3, WNT1, YY1AP1, ZNF687*. Sequencing was performed on a NextSeq 500 platform (Illumina, San Diego, CA, USA) using the MidOutput flow cells (300 cycles) with a minimum expected coverage depth of 200X. For filtering, prioritisation, interpretation of single-nucleotide variants and copy number variations, we followed an internal laboratory-defined adaptation of the worldwide consensus guidelines of the ACMG (S1). MLPA Kit (P484-ALPL, MRC Holland) was applied on negative subjects and carried out following the manufacturer’s instructions.

Cell lines. Assays were carried out on HEK293 cells (ECACC) that do not express an endogenous TNSALP (S2, S3). Cells were grown at 37 °C, 5% CO_2, in DMEM + Glutamax (GIBCO, Thermo-Fisher, Waltham, MA, USA) supplemented with 10% FBS (GIBCO) and 1% Penicillin Streptomicin (Sigma-Aldrich, Sant Louis, MO, USA).

Vectors. The different variants were introduced in a basic pcDNA3.1 vector containing the wild type ALPL sequence and encoding for the native protein, by classic site-specific mutagenesis protocol and the correctness of the mutated vectors verified through colony PCR and Sanger sequencing. As internal control, two known deleterious variants were included [p.(Tyr28Asp) for the Western Blot and the p.(Ser310Profs*28) (S4) for the colorimetric assay].

Western Blot. HEK293 were seeded in 6 well plates, transfected with 1.5 ug DNA of WT/Mut ALPL using Lipofectamine 3000 Transfection reagent (ThermoFisher), following the manifacturer’s instructions. 48 h after, whole crude protein lysate extracts were obtained in RIPA buffer [150 mM NaCl, 50 mM Tris-HCl,

1%, Nonidet P-40, 0.1% sodium dodecyl sulfate (SDS), 0.5% sodium deoxycholate, pH 8.0] + with Complete EDTA, phosphatase/protease inhibitor and PhosStop (both from Roche, Mannheim, Germany, both 1 tablet/10 mL). Proteins were denaturated at 70 °C x 10', loaded onto a 10% SDS polyacrylamide gel, electrotransferred (Transblot, Biorad, Hercules,CA,USA), blotted overnight at 4°C with primary monoclonal antibody (1:200, anti-TNAP, sc137213, Santa Cruz Biotechnology, Dallas,TX, USA) or reference (1:200, beta-actin, sc-517582, Santa Cruz Biotechnology). Membranes were blotted for 1 h at RT with secondary antibody (1:7000, goat anti-Mouse, Biorad). Chemiluminescent reaction was done with Pierce (ThermoFisher) and signal detection through the Chemidoc System (Biorad).

Colorimetric Assay. HEK293 cells were seeded in 96 wells plates, transfected in eight replicates with 75 ng of WT/Mut vectors. After 48 h, Residual Enzymatic Activity (REA) was measured by the Amplite Colorimetric Alkaline Phosphatase Assay Kit (Yellow Color, AAT Bioquest, Pleasanton, CA, USA) following the manufacturer's instructions.

In silico analysis. Atomic coordinates of the ALPL protein in its dimeric form were obtained using the specialized AlphaFold-multimer model (S5, S6). Structural changes induced by missense variants were deduced by the Missense3D web tool (S7), that provides an estimate of the structural impact of the variant. Stability of each mutant structure was investigated thermodynamically through the BuildModel function implemented in the FoldX algorithm (S8) run with standard parameters.

FoldX computed the total energy of both monomeric and dimeric ALPL protein, wild-type and mutated, as a proxy of their overall stability and the Van der Waals interresidue clashes, as energy penalization factors. Thus, both pdb structures were minimized, before assessing their stability, namely all the side chains were slightly moved in order to reduce the Van der Waals' clashes. Mutations were classified as destabilizing when $\Delta\Delta G$ exceeded +0.61 kcal/mol for the monomer and +1.2 kcal/mol for the dimer. Conversely, mutations were considered stabilizing when $\Delta\Delta G$ values were below −0.61 kcal/mol (monomer) and −1.2 kcal/mol (dimer) (Table 2).

Statistical analysis. Signal densitometry of the TNSALP 88/66 kDa bands was calculated (Image Lab software, Biorad) and data expressed as mean and standard deviation derived from triplicates experiments with a p-value < 0.05 for statistical significance.

ID	Sex	Age (ys)	AaD (ys) first symptoms	Delay in disease recognition (ys)	ALP (range, UI)	Vit B6 (7 – 18 μg/mL)	Symptomatic Asymptomatic Atypical	Clinical symptoms	HPP Classification
#1-1 daughter	F	38	38	38	20 (35-104)	73	Asymptomatic	none	NAp
#1-2 daughter	F	41	41	41	21 (35-104)	42	Asymptomatic	none	NAp

Suppl Table 1. Clinical and biochemical data of two relatives, daughters of #1.

Supplemental references:

1. Richards S, Aziz N, Bale S, et al. Standards and guidelines for the interpretation of sequence variants: a joint consensus recommendation of the American College of Medical Genetics and Genomics and the Association for Molecular Pathology. Genet Med. (2015) 17:405–424. doi: 10.1038/gim.2015.30

2. Hofmann C, Liese J, Schwarz T, et al. Compound heterozygosity of two functional null mutations in the ALPL gene associated with deleterious neurological outcome in an infant with hypophosphatasia. Bone. (2013) 55:150–157. doi: 10.1016/j.bone.2013.02.017

3. Yang H, Wang L, Geng J, et al. Characterization of six missense mutations in the tissue-nonspecific alkaline phosphatase (TNSALP) gene in Chinese children with hypophosphatasia. Cell Physiol Biochem. (2013) 31:635–644. doi: 10.1159/000354467

4. Whyte MP, Zhang F, Wenkert D, et al. Hypophosphatasia: validation and expansion of the clinical nosology for children from 25 years experience with 173 pediatric patients. Bone. (2015) 75:229–239. doi: 10.1016/j.bone.2015.02.022

5. Evans R, O'Neill M, Pritzel A, et al. Protein complex prediction with AlphaFold-Multimer. bioRxiv. (2021). doi: 10.1101/2021.10.04.463034

6. Mirdita M, Schütze K, Moriwaki Y, et al. ColabFold: making protein folding accessible to all. Nat Methods. (2022) 19:679–682. doi: 10.1038/s41592-022-01488-1

7. Ittisoponpisan S, Islam SA, Khanna T, et al. Can predicted protein 3D structures provide reliable insights into whether missense variants are disease associated? J Mol Biol. (2019) 431:2197–2212. doi: 10.1016/j.jmb.2019.04.009

8. Schymkowitz J, Borg J, Stricher F, et al. The FoldX web server: an online force field. Nucleic Acids Res. (2005) 33:W382–W388. doi: 10.1093/nar/gki387
